# Medicine Through the Lens of Humanity: Exploring the Legacy of Oliver Sacks, the Storyteller Neurologist

**DOI:** 10.7759/cureus.69593

**Published:** 2024-09-17

**Authors:** Tal Sharon, Zhao Xiang Lin, Julio Palomera, Sara Jaime, Anita Szerszen

**Affiliations:** 1 Medical School, Touro College of Osteopathic Medicine, New York, USA; 2 Geriatrics, Northwell Health, Staten Island, USA

**Keywords:** historical vignette, lgbt, medical pioneer, neurology, palliative care

## Abstract

In an era of medicine when a paternalistic approach was the norm, neurologist Dr. Oliver Sacks infused his care with humanity, centering a dialogue that emphasized his patients’ perspective and personhood. For most of his life, he encountered significant personal challenges, and struggled with prosopagnosia and an internal conflict with his sexuality. Instead of allowing these challenges to hold him back, he transformed them into the driving force behind a career that uniquely combined the scientific beauty of neurology and a humanistic patient-centered approach with the power of literature. Through non-fiction works like *Awakenings* and *The Man Who Mistook His Wife for a Hat*, he translated the overwhelming complexities of neurological disorders into stories that touched the hearts of readers around the world, making the science of the brain more accessible and human. In his later years, Dr. Sacks grappled with his mortality and the broader questions of aging and life’s purpose, themes central to his final book, *Gratitude*. His reflections emphasized the importance of aligning healthcare with patients' personal values and goals. Dr. Sacks’ legacy is marked not only by his professional contributions and literary achievements but also by his deep compassion and innovative approach to patient care, which continues to influence modern medical practice and inspire an ongoing discourse about the intersection of science, humanity, and individual experience.

## Introduction and background

**Figure 1:**
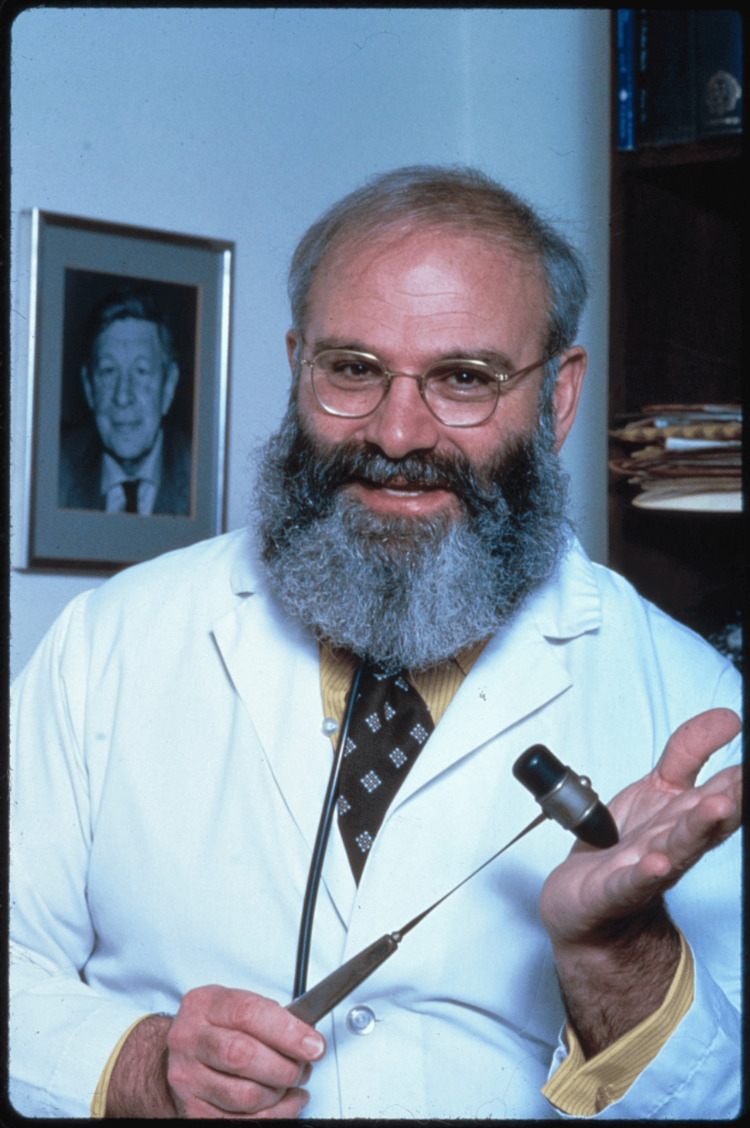
Portrait of Dr. Oliver Sacks Photo credit: Bernard Gotfryd, Library of Congress. There are no known copyright restrictions on Bernard Gotfryd's photographs in Library of Congress collections. Mr. Gotfryd's photographs were restricted during his lifetime. Mr. Gotfryd died in 2016 [6].

Dr. Oliver Sacks (Figure 1) introduced a humanistic approach to the practice of neurology by channeling his personal challenges into profound empathy. He prioritized the unique narratives and personhood of every patient he encountered, writing about their experiences during a time when medicine focused primarily on diseases rather than on people living with them.

Dr. Sacks was born into a family of physicians in London in 1933 [1]. He was a quiet child, deeply immersed in his thoughts, yet often battling feelings of isolation [2]. His struggle with prosopagnosia, a condition that made it difficult for him to recognize faces, along with his homosexuality during a time when it was punishable by law, only intensified this sense of loneliness [1,3]. Though perhaps it may have been these very struggles that propelled his desire to humanize his patients, a shift now recognized as a foundation of patient-centered care [1].

Science, especially chemistry, was his escape. Influenced by his family and growing desire to connect with people more directly, Dr. Sacks pursued a medical degree at Oxford University, which he completed in 1958 [1,2]. He continued his medical education in the United States; first at Mt. Zion Hospital in San Francisco, then at UCLA where he completed his residency in neurology and neuropathology [1].

In 1966, while working as a neurologist at Beth Abraham Hospital in the Bronx, Dr. Oliver Sacks encountered 80 patients who had been in catatonic states for over 50 years. Their catatonia and immobility were consequences of the encephalitis lethargica, a mysterious parkinsonism-like illness that had swept through the early 20th century, for which the cause was never found [4]. An astute observer and thinker, Dr. Sacks used the experimental drug L-dopa, which temporarily alleviated the post-encephalitis stupor, a profoundly moving experience that he later recounted in his book, *Awakenings* [4].

Throughout his career, Dr. Sacks studied a wide range of neurological disorders, including Tourette’s syndrome and phantom limb syndrome, always with a focus on the unique and deeply personal narratives of each patient he encountered [3]. Dr. Sacks earned worldwide recognition as a masterful storyteller, seamlessly combining scientific knowledge, and clinical observations with literary elegance [1,5]. His unique ability to convey the complexities of the human brain in a way that was both accessible and deeply moving earned him the title of "poet laureate of medicine" [1]. Though his work was mostly dismissed by the more traditional scientific community, he was widely loved by the public and became an influential figure in the world of narrative medicine and neurology.

Despite early skepticism, Dr. Sacks’ contributions were recognized through numerous awards, including the prestigious Lewis Thomas Prize and several honorary degrees [1]. He was appointed a Commander of the British Empire for his services to literature and medicine, and a few years before his death, named a Fellow of the American Academy of Arts and Sciences.

His essays on life, death, and the complexities of the human experience have left a lasting legacy that continues to resonate in both the medical community and beyond [5]. Within the fields of neurology, geriatrics, and psychology, Sacks may be seen as a pioneer in promoting patient-centered care and the importance of viewing patients holistically, not just as a set of symptoms or a diagnosis.

Dr. Oliver Sacks' legacy endures, not only through his prolific written work but also in the deep empathy and insight he shared with both his patients and his readers [1,5].

## Review

Struggles with sexual identity

Dr. Oliver Sack’s life serves as a reminder of the importance of authenticity and self-acceptance. As a gay man, Dr. Oliver Sacks faced significant struggles with his sexual identity throughout his life. His journey towards self-acceptance and understanding his identity was complex, shaped by hostile societal pressures, his family, and his personal experiences. These internal struggles often influenced his clinical work, and consequently, his writing. He felt a deep empathy for those marginalized or misunderstood, and through the lens of empathy was able to tell their stories.

Dr. Sacks only began discussing his sexuality publicly later in life, but from an early age, he grappled with accepting his identity, an internal struggle vividly recounted in his memoir, *On the Move: A Life *[1]. A pivotal moment in his life occurred when his father disclosed his sexuality to his mother against his wishes, leading her to call him an "abomination” [1]. This painful interaction deeply affected Dr. Sacks, contributing to the guilt and inhibition that restrained him from fully embracing his identity for many years.

During his time in San Francisco in the 1960s, Dr. Sacks faced discrimination and internalized homophobia, leading him to use substances as a form of self-medication. In *Hallucinations*, he describes a turning point in his life: the satisfaction he discovered in writing about his patient experiences [7]. He noted that the joy he derived from writing was "infinitely more substantial than the vapid mania of amphetamines," marking the beginning of his path to sobriety [7].

After remaining celibate for 35 years due to fears about the impact of his sexuality on his career, Dr. Sacks found love with Bill Hayes in 2008. They stayed together until Dr. Sacks' death in 2015. In his memoir, *On the Move: A Life*, Dr. Sacks admitted to having "lived at a certain distance from life," [1] keeping certain emotions at bay for self-preservation. Meeting Hayes and falling in love allowed Dr. Sacks to break free from a lifetime of solitude and fully embrace his true self. These personal struggles deeply influenced his medical practice, instilling in him a profound compassion and curiosity. His experiences led him to approach his patients with empathy and compassion, in his writing to focus on humanistic aspects of medicine, reflecting his own journey through adversity.

Encephalitis lethargica

In *Awakenings*, Dr. Sacks describes his work with a group of patients who had been deemed incurable after suffering from the encephalitis lethargica epidemic of 1916-30, a “sleepy sickness”. These patients were described as “human statues”, trapped in a coma-like state, rigid and speechless [4]. Encephalitis lethargica was classified into two phases: the acute phase, marked by excessive sleepiness, fever, movement disorders, and disorders of ocular motility, and the chronic phase distinguished by parkinsonian-like signs [8]. There were three different forms of this disease based on the symptom array: somnolent-ophthalmoplegic, hyperkinetic, and amyostatic-akinetic, with the somnolent-ophthalmoplegic being the most common [8]. Patients often appeared dazed and confused, exhibiting features of mild meningeal irritation [8]. Notably, patients experienced a strong desire to sleep and would be found sleeping for long periods of time, though they remained aware of their surroundings while asleep [8].

Dr. Sacks “awakened” these patients when he administered the experimental drug, L-dopa, the “remarkable new ‘awakening’ drug” [4]. He described the most severely affected patients as “extinct volcanoes” and observed that after receiving L-dopa, they “erupted into life” after being regarded as effectively dead for so long [4]. Though the effects were short in nature, this discovery, ignited significant interest in the usage of L-dopa for other neurological conditions. It also stimulated further research into the pathophysiology of neurological conditions and the potential for pharmacological intervention [8].

After *Awakenings* gained worldwide recognition, it was adapted into a film in 1990, in which Robin Williams portrayed Dr. Sacks. This film not only enlightened the public on this epidemic and the dramatic effects of L-dopa, but also humanized medicine [8]. 

“Poet laureate of contemporary medicine”

Dr. Oliver Sacks, a renowned neurologist and a best-selling author, is celebrated for his books and articles published by the New York Times, that inspired various adaptations, such as a movie, a play, and an opera. He is best known for his case histories such as *The Man Who Took His Wife for a Hat: and Other Clinical Tales*, *Musicophilia: Tales of Music and the Brain*, *An Anthropologist on Mars*, and *Awakenings.* He also published many pieces in the New York Times on religion, hobbies, and his personal life. His last publication, “Sabbath”, was published in the New York Times on August 14th, 2015, just a few days before he passed away [9]. More of Dr. Sacks' notable publications and adaptations of his work can be found in Appendix 1. 

On death and dying

Dr. Oliver Sacks’ final book, *Gratitude*, provides a profound perspective on aging, mortality, and the art of living [10]. Dr. Sacks delved into deep self-reflection as he embraced his final years, his writings offering a powerful perspective on how healthcare can better serve patients by focusing on what truly matters to them.

In *Gratitude*, his essay "Mercury," Dr. Sacks contemplates the significance of turning 80, describing the mental clarity and broadened perspective that come with age [10]. He speaks to the richness of life experiences that inform this stage of life, allowing one to appreciate both beauty and impermanence. His emphasis on understanding and honoring the goals of care in older patients spoke to his mission to center medicine on a patient’s personal values and life goals [10].

Dr. Sacks’ reflection in *Gratitude* written after his terminal cancer diagnosis, illustrates the importance of living with intention [10]. He chose to focus on deepening relationships and continuing his work, while also letting go of concerns that no longer served his life's purpose. Dr. Sacks’ poignant reflections in *Gratitude* serve as a powerful reminder of the need for healthcare providers to engage in meaningful conversations about goals of care, especially as patients face the end of life. His work underscores the importance of empathy, understanding, and the human connection in medicine, principles that shed light on personhood and humanity for patients.

Legacy

Dr. Sacks’ extensive publications in the field of neurology helped public understanding of syndromes like Tourette’s and Asperger’s. Through his literary voice, he humanized these patients, sharing their histories and struggles. From retinitis pigmentosa and the world of people living with hearing loss to hallucinations and schizophrenia, he wrote about his patients’ experiences, and by doing so, gave people more insight into the confounding neurological disorders of that time. In his novel, *Musicophilia: Tales of Music and the Brain*, Dr. Sacks explored the connection between music and the brain, advocating for the use of musical therapy for patients with neurological conditions like Parkinson’s disease [11]. What distinguishes Dr. Sacks from his contemporaries is his unique approach to documenting his patients' experiences and probing into the psychological dimensions of disease pathology, an approach now recognized as narrative medicine. He explored a profound intersection between the complexities of the human mind and the disorders that challenge it. He answered the questions many of us ponder: can music heal our minds? How do we define our physical identity? Can we live a purposeful and meaningful life? [10-12]

His passion for neuroscience and medicine extended beyond his role as a physician; he delved into the ideas and controversies surrounding all of science. Dr. Sacks will be remembered not just for his accomplishments as a neurologist and writer, but for his unconventional and innovative approach to medicine. He did not try to fit his patients into a preconceived box, instead, he embraced their individuality, tailoring his approach to each person’s unique way of experiencing the world, embodying true patient-centered care.

## Conclusions

Dr. Oliver Sacks' influence on both the medical and literary worlds is profound and enduring. His ability to weave together clinical detail with human experience brought a unique depth to the understanding of neurological disorders, and his writings have inspired countless individuals to view medicine through a more empathetic lens. His work continues to influence modern medicine, especially in the growing emphasis on personalized and compassionate care. His legacy lies not just in his scientific and literary contributions, but in the way he elevated the stories of his patients, giving a voice to those often overlooked by society.

## Appendices

Appendix 1

Dr. Sacks also published many pieces in the New Yorker on religion, hobbies, and his personal life. Table 1 shows a chronological timeline of this collection. 

**Table 1:** A chronological timeline of some of the most famous works of Dr. Oliver Sacks.

Category	Title	Year
Books [13]	Awakenings	1973
	A Leg to Stand On	1984
	The Man Who Mistook His Wife for a Hat: and Other Clinical Tales	1985
	Seeing Voices	1989
	An Anthropologist on Mars	1995
	The Island of the Colorblind	1997
	Uncle Tungsten: Memories of a Chemical Boyhood (autobiography)	2001
	Musicophilia: Tales of Music and the Brain	2007
	The Mind’s Eye	2010
	Hallucination	2012
	On the Move: A Life (autobiography)	2015
	Gratitude	2015
	The River of Consciousness	2017
	Everything in Its Place: First Loves and Last Tales	2019
Literary Works [14]	“A surgeon’s life”	1992
	“Sudden sight, after a lifetime of blindness”	1993
	“An Anthropologist on Mars”	1993
	“The Case of Anna H.”	2002
	“The Mind’s Eye”	2003
	“A Man of Letters”	2010
	“Face-Blind”	2010
	“The Catastrophe”	2015
Scientific articles	“Effects of levodopa in parkinsonian patients with dementia” [15]	1972
	“The power of music” [16]	2006
Adaptations based on works by Oliver Sacks [13]	“A Kind of Alaska” (play)	1982
	Awakenings (movie)	1990
	Oliver Sacks: His Own Life (documentary)	2019
	Awakenings (opera)	2022
	Radiant Minds: The World of Oliver Sacks (podcast)	2022
